# Atrial electrogram differences between a dual-energy ablation catheter and a conventional mapping catheter

**DOI:** 10.1016/j.hroo.2025.09.008

**Published:** 2025-09-13

**Authors:** Vincenzo Mirco La Fazia, Elio Zito, Sanghamitra Mohanty, Carola Gianni, Giuseppe Stifano, J. David Burkhardt, Rodney Horton, Amin Al-Ahmad, Luigi Di Biase, Andrea Natale

**Affiliations:** 1 Texas Cardiac Arrhythmia Institute, St David's Medical Center, Austin, Texas; 2 Division of Cardiology, Department of Biomedicine and Prevention, University of Tor Vergata, Rome, Italy; 3 Department of Electrophysiology, Albert Einstein College of Medicine, New York, New York; 4 Interventional Electrophysiology, Scripps Clinic, San Diego, California; 5 Metro Health Medical Center, Case Western Reserve University School of Medicine, Cleveland, Ohio

**Keywords:** Atrial fibrillation, Catheter ablation, Pulsed field ablation, Mapping system, Electrograms

**Key Findings**

▪ During mapping of the pulmonary veins (PVs) and posterior wall (PW), electrogram (EGM) quality was comparable between Sphere-9 and Lasso.
▪ In areas outside PVs and PW, Lasso produced EGMs with higher amplitude and more defined deflections than Sphere-9.
▪ Operators should be cautious interpreting Sphere-9 signals outside PVs and PW because they may differ from standard mapping catheters because of differences in electrode size/spacing, catheter–tissue contact, and local substrate characteristics.

## Introduction

Pulse field (PF) ablation (PFA) has emerged as a novel non-thermal energy source for atrial fibrillation (AF) ablation. Owing to its high tissue selectivity and penetrance, PF has demonstrated better safety compared with thermal energy.^1^ However, most PFA systems lack mapping features. The Sfere-9™ is the first Food and Drug Administration-approved catheter integrated with a mapping system (Affera, Medtronic Inc) and capable of delivering both radiofrequency and PF energy.^2^

The aim of our study was to compare the signals detection properties of the Sphere-9 catheter with a diagnostic multipolar mapping catheter (LASSO™, Biosense Webster).

We analyzed prospectively collected data from consecutive AF patients undergoing PFA from November 2024 to February 2025. Before the procedure, each patient provided written informed consent to undergo the ablation procedure. The study received approval from the local Ethics Committee and was conducted in accordance with the Declaration of Helsinki. Patient-level data was collected in the Institutional Review Board-approved institutional databases.

After intracardial echocardiogram-guided transeptal access, the Sfere-9 catheter was inserted in the left atrium for electroanatomical mapping. The ablation catheter was then removed, and a 20-pole LASSO was inserted in the left atrium to compare the signal in pulmonary veins (PVs), posterior wall (PW), mitral isthmus, roof, and left atrial appendage. The signals were recorded in a bipolar configuration using the Prucka 3 with CardioLab™ digital recording electrophysiology system (GE Healthcare). Electrograms (EGMs) were filtered with a bandpass range of 30–500 Hz, using a gain setting of 5000. A qualitative assessment of amplitude (mV) and duration (msec) of each signal was performed. Accurate position and contact of the ablation and the mapping catheter were confirmed with both fluoroscopy and intracardial echocardiogram. 2 independent operators analyzed the signals, and a third opinion was taken in the event of disagreement between the 2 experts.

**Figure 1:**
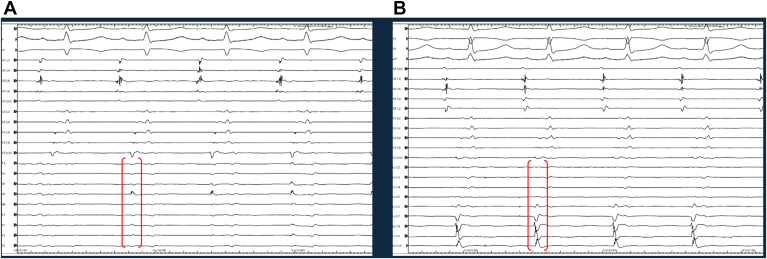
Electrograms obtained from the LASSO in the perimitral area. **A:** Showed a better sharpness, more defined deflections and higher amplitude when compared with those collected with the Sphere-9 catheter in same location (**B**).

A total of 35 patients (30, 85.7% men) with a median age of 70 years (interquartile range 65–76 years) and a persistent AF rate of 82.7%. At the beginning of the procedure, 20 patients (57.1%) were in sinus rhythm, 8 (22.9%) in atypical atrial flutter, and 7 (20%) in AF. The quality of EGMs recorded from both catheters was comparable during mapping of the PVs and PW. However, in the remaining anatomical regions (mitral isthmus, left atrial appendage, and roof), EGMs obtained with the LASSO catheter were superior in terms of sharpness (Figure 1).

Variations in signals characteristics can typically be due to multiple factors, including electrode size and spacing, catheter-tissue contact, filtering settings, and tissue properties (eg, presence of scar or fibrosis).^3^ The LASSO is a circular mapping catheter with 20 electrodes, each 1 mm, spaced 2-6-2 mm. In contrast, the Sphere-9 features a collapsible 9-mm spherical surface with 9 0.7 mm electrodes, each individually coupled to a central reference one. Despite the increasing use of PFA, which has been associated with a reduction in ablation-related complications, data from the MANIFEST-REDO study have shown a high rate of reconnections, presumably because of the absence of a dedicated mapping system.^4^ The Affera system may help address this limitation by integrating both mapping and ablation capabilities.

Operators should be aware of EGM quality, particularly in areas outside the PVs and PW, as the signals may differ significantly from those typically observed with standard mapping catheters.

## Disclosures

Dr Di Biase is a consultant for Biosense Webster, Boston Scientific, Stereotaxis, and St. Jude Medical, and has received speaker honoraria from Medtronic, Atricure, EPiEP, and Biotronik. Dr Natale is a consultant for Abbott, Biosense Webster, Biotronik, Boston Scientific, Medtronic, and iRhthym. All other authors have reported that they have no relationship relevant to the contents of this paper to disclose.
